# Menière’s disease clinical subtypes and baseline characteristics in a Dutch patient cohort

**DOI:** 10.1007/s00405-026-10218-8

**Published:** 2026-05-05

**Authors:** Maud M.E. Boreel, Hester S. Verhoeven, Babette F. van Esch, Tjard R Schermer, Peter Paul G. van Benthem, Tjasse D. Bruintjes

**Affiliations:** 1 Department of Otorhinolaryngology—Head and Neck Surgery, Leiden University Medical Center, Leiden, The Netherlands; 2 Apeldoorn Dizziness Centre, Gelre Hospital, Apeldoorn, The Netherlands

**Keywords:** Menière’s Disease, Clinical subtypes, Unilateral and bilateral Menière’s disease, Retrospective cohort study

## Abstract

**Purpose:**

The aim of this study is to provide a detailed description of a Dutch cohort of patients with Menière’s disease (MD), classify patients into clinical subtypes according to the criteria proposed by Frejo et al., and compare the distribution of these subtypes with cohorts from Spain, the United States, and China.

**Methods:**

A retrospective, cross-sectional chart review was conducted among 375 patients diagnosed with definite MD at two Dutch medical centers. Patients were classified into five clinical subtypes of unilateral MD or 5 subtypes of bilateral MD. Baseline characteristics were analyzed and compared across subtypes and international cohorts.

**Results:**

Of the 375 included patients, 335 had unilateral MD of which 49.0% were type 1 (classic MD), 23.6% type 2 (delayed MD), 6.6% type 3 (familial MD), 13.7% type 4 (migraine-associated MD), and 7.2% type 5 (autoimmune MD). This distribution differed significantly from the Spanish cohort regarding the delayed and familial subgroups, and from the Chinese cohort regarding the familial subgroup. Forty cases of bilateral MD were observed, with differences in the synchronic subgroup when compared to the Spanish cohort.

**Conclusion:**

This study describes the clinical subtypes of MD within a Dutch patient cohort, with subtype distributions more closely resembling those of the American and Chinese cohorts than the Spanish cohort. This suggest regional variation in the clinical expression of MD.

## Introduction

Menière’s Disease (MD) is an inner ear disorder characterized by recurrent vertigo attacks, sensorineural hearing loss, tinnitus and aural fullness [1]. While in most patients with MD endolymphatic hydrops can be established through MRI-scanning, the exact pathophysiology of the disease remains unknown [2]. It is thought that multiple factors including genetic, viral, allergy and autoimmunity factors may contribute to the development of MD [3]. A recent prospective study by Frejo et al. identified three distinct clusters: autoinflammatory (characterized by high levels of IL1B), allergic (marked by elevated IgE, IL4, IL10, and IL13), and autoimmune (with low cytokine levels) [4]. These findings suggest that different inflammatory mechanisms may contribute to the development of MD.

The diagnosis is made by the presence of the abovementioned characteristic clinical symptoms [1]. However, these criteria do not take into account the clinical heterogeneity of the disease. Symptoms of MD manifest with considerable variability, with the classic triad of episodic vertigo, sensorineural hearing loss, and tinnitus occurring simultaneously at disease onset in approximately 40% of cases [5]. When we can identify different clinical subtypes of MD with potential different pathogenesis, clinical trials on the treatment of MD can be more personalized and specific, making these treatments potentially more effective [3].

Frejo et al. conducted a multicenter cross-sectional study on patients with unilateral MD, distinguishing between sporadic cases of MD, which lack a known family history and familial cases, defined by the presence of a first- or second-degree relative meeting the criteria for definite or probable MD [6]. In this cohort 824 unilateral MD patients and 398 bilateral cases were analyzed and they defined five subgroups for unilateral MD: classic MD, delayed MD, familial MD, migraine associated MD and autoimmune MD [6, 7]. Crossley et al. identified these clinical subtypes for a US-based cohort of 81 patients and found a different distribution of unilateral MD subtypes compared to the population of Frejo et al. [8]. In China, Chen et al. analyzed the clinical subtype distribution of unilateral MD in a cohort of 245 patients and subsequently evaluated the therapeutic efficacy of various vertigo treatments per subgroup over a 24-month follow-up period [9]. Their results were more comparable to the findings reported by Crossley et al. then those of Frejo et al.

This study evaluates a Dutch cohort of patients with Menière’s disease, outlines baseline clinical characteristics, and examines the distribution of unilateral and bilateral MD phenotypic subgroups, which are compared with those reported by Frejo et al., Crossley et al., and Chen et al. Comparing subtype distributions between countries may provide insight into population-based differences in disease presentation and helps to evaluate the generalizability of existing MD phenotypic classifications.

## Methods

This retrospective, cross-sectional, chart review, examined medical records of patients diagnosed with definite MD, as defined by the American Academy Otolaryngology-Head and Neck Surgery, Classification Committee of the Bárány Society, European Academy of Otology and Neurotology and International Classification of Vestibular Disorders, published in 2015 [1]. Patients were recruited from two medical centers in the Netherlands: the Apeldoorn Dizziness Centre (ADC) and the Leiden University Medical Center (LUMC). The sample size was calculated to ensure representativeness of the Dutch MD population, estimated at approximately 15,000 cases (0.6–1.0 per 1,000 people) [10]. Ethics approval for the study was obtained from both institutions.

Medical records were reviewed to determine whether patients met the inclusion criteria. Eligible patients were those diagnosed with either unilateral or bilateral definite MD, aged over 18 years, and a recorded diagnostic code for MD between 2015 and 2024. We collected clinical data related to the subgroup classification defined by Frejo et al. [6, 7]. Furthermore, secondary endpoints focused on patient characteristics including age, weight, height, gender, disease duration, hearing loss at diagnosis, and the most recent hearing assessment (pure tone audiometry [PTA] and word recognition score [WRS]). Additional factors included hearing loss stage, cardiovascular risk factors (hypertension, dyslipidaemia, type 2 diabetes, smoking, and BMI), the presence of Tumarkin drop attacks, MD treatments, and alternative diagnoses. If the required data was not fully available in medical records, patients were contacted by telephone to obtain the missing information.

Subsequently, all patients were classified into five distinct subgroups for unilateral MD, and five subgroups for bilateral MD, as described in the studies by Frejo et al. (Table 1) [6, 7]. For unilateral MD, type 1, classic MD, was defined as definite MD without a history of migraine, delayed MD, or autoimmune disorders. Type 2, delayed MD, included patients with a history of sensorineural hearing loss (SNHL) occurring at least one month before the onset of vertigo. Patients were asked whether their sensorineural hearing loss (SNHL) had been confirmed by an audiogram, as self-reported hearing loss without objective verification was not considered adequate. Type 3, familial MD, was characterized by the presence of a first- or second-degree relative diagnosed with MD by a physician (delayed MD excluded). Type 4, migraine-associated MD, included sporadic patients who were diagnosed with migraine or migraine with aura by a physician, without delayed MD. Finally, Type 5, autoimmune MD, encompassed sporadic patients diagnosed with both MD and an autoimmune disease by a physician, including all conditions with a known autoimmune etiology such as rheumatoid arthritis, colitis, celiac disease, Graves’ disease, and Hashimoto’s thyroiditis, without delayed MD or a history of migraine. A similar classification was made for bilateral MD, with a distinction between metachronic and synchronic hearing loss. Metachonic hearing loss describes symptoms that appear in one ear before the other while synchronic hearing loss refers to the simultaneous onset of symptoms in both ears.

**Table 1:** Definitions of Menière’s disease clinical subtypes for unilateral and bilateral MD, as defined by Frejo et al

Subgroup	Definition
Unilateral MD	
Type 1 “Classic MD”	Patients with sporadic MD, without migraine, delayed MD or auto Immune disorders
Type 2 “Delayed MD”	History of SNHL ≥ 1 month prior to vertigo onset
Type 3 “Familial MD”	Family history of MD, without delayed MD
Type 4 “Migraine MD”	Patients with sporadic MD and a history of migraine, without delayed MD
Type 5 “Auto Immune MD”	History of autoimmune disease, without migraine or delayed MD
Bilateral MD	
Type 1 “Metachronic MD”	SNHL, without migraine, familial MD or autoimmune disease
Type 2 “Synchronic MD”	Synchronic onset of MD without autoimmune disease
Type 3 “Familial MD”	Family history of MD, without autoimmune disease
Type 4 “Migraine MD”	History of migraine and ‘sporadic MD’
Type 5 “Auto Immune MD”	All patients with bilateral MD and autoimmune disease

*MD* Menière’s disease, *SNHL* sensorineural hearing loss

### Statistical analysis

Descriptive analyses were conducted on the data of all patients included in our study cohort and are presented as means with standard deviations or as counts with percentages. ANOVA was used to assess statistically significant differences in continuous and interval demographic and clinical variables across subgroups. When statistically significant differences were found, post-hoc comparisons were conducted using Tukey’s HSD test. A Bonferroni correction was applied where appropriate, to control for multiple comparisons. To compare categorial variables between subgroups, cross-tabulations and chi-square tests were performed. To evaluate whether there were significant differences in the distribution of unilateral Menière’s disease subgroups between the current study and those reported by Frejo et al., Crossley et al., and Chen et al., Pearson chi-square goodness-of-fit tests were used. Due to the small sample size in Crossley’s et al. bilateral MD cohort, a Fisher exact test was applied for the comparison of the subgroup distribution in this cohort to our bilateral group. Additionally, unpaired t-tests and Pearson’s chi-square tests were conducted to compare demographic and clinical characteristics of the unilateral sporadic MD populations between the current study and the other studies. A p-value of less than 0.05 was considered statistically significant. Statistical analyses were conducted using SPSS software, version 28 or later (SPSS, Chicago, Illinois, USA).

## Results

A total of 632 medical records were reviewed, resulting in the inclusion of 375 patients with definite MD, 96 from LUMC and 279 from ADC. Of these patients, 182 (48.5%) were female, the average age of onset was 53.9 (SD 14.7) years, 40 (10.7%) had bilateral MD, and 85 (22.7%) had experienced a Tumarkin drop attack at some point in their lives (Table 2). Two hundred eighty patients (83.6%) received treatment for MD. Among them, 202 (60.3%) used betahistine, 67 (20.0%) used cinnarizine, 107 (31.9%) had received intratympanic corticosteroid injections, and 16 (4.8%) had received gentamicin injections. Additionally, 4 (1.2%) patients had undergone surgery, 22 (6.6%) had received physiotherapy, and 20 (5.3%) used prisma goggles. Other treatments reported included diuretics, ventilation tubes, acupuncture, bioresonance, and homeopathic remedies.

**Table 2:** Characteristics of unilateral and bilateral cases of Menière’s disease in the current cohort

	Both groups	Unilateral MD	Bilateral MD	
Cases, *n*	375	335		40
Gender, n (% women)	182 (48.5 )	170 (50.7)		12 (30)
Unilateral, n (%)	335 (89.3)	-		-
Affected side (unilateral), n (% left)	185 (55.2)	185 (55.2)		-
Age of onset, mean (SD)	53.9 (14.7)	54.6 (14.6)		48.2 (14.7)
Delayed MD, n (%)	89 (23.7)	79 (23.6)		10 (25)
Family member with MD, n (%)	33 (8.8)	29 (8.7)		4 (10)
Migraine, n (%)	68 (18.1)	61 (18.2)		7(17.5)
Autoimmune disease, n(%)	61 (16.3)	53 (15.8)		8 (20)
Cardiovascular risk				
BMI, mean (SD)	25.75 (6.7)	25.57 (6.7)		27.0 (7.2)
High blood pressure, n (%)	138 (36.8)	125 (37.3)		13 (32.5)
Dyslipidemia, n (%)	118 (31.5)	103 (30.7)		15 (37.5)
Type 2 diabetes, n (%)	27 (7.2)	25 (7.5)		2 (5)
Smoking, n (%)	55 (14.7)	46 (13.7)		9 (22.5)
Tumarkin crisis, n (%)	85 (22.7)	72 (21.5)		13 (32.5)
Anxiety or depression, n (%)	80 (21.3)	71 (21.2)		9 (22.5)

Baseline characteristics of all cases included in the current study. *SD* standard deviation, *MD* Menière’s disease

**Fig. 1:**
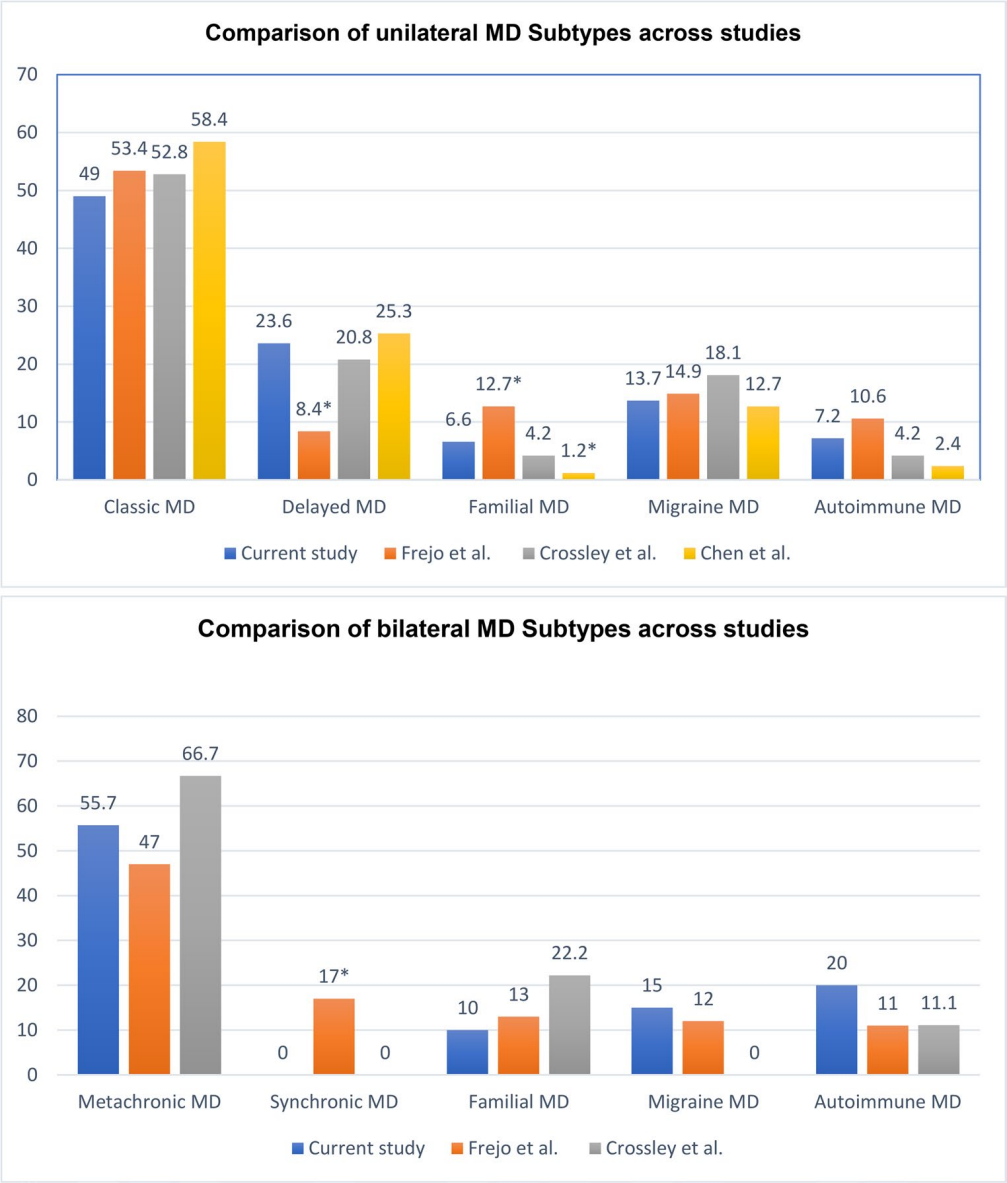
Comparison of unilateral and bilateral Menière’s disease subgroups across studies. MD = Menière’s disease; * indicates a statistically significant difference between the subgroup in the current study and those in the comparison studies. The study by Chen et al. did not examine bilateral MD

Figure 1 shows the distribution of subtypes in our cohort in comparison with the three studies previously mentioned [6, 8, 9]. Among the 335 patients with unilateral MD 164 (49.0%) were classified as classic MD, 79 (23.6%) as delayed MD, 22 (6.6%) as familial MD, 46 (13.7%) as migraine-associated MD, and 24 (7.2%) as autoimmune MD. The cohort of bilateral MD in our study consisted of 40 patients: 22 (55.7%) were classified as metachronic MD, none as synchronic MD, 4 (10.0%) as familial MD, 6 (15.0%) as migraine-associated MD, and 8 (20.0%) as autoimmune MD.

Of the unilateral MD patients, 29 patients had a first or second degree family member diagnosed with MD by a physician, for one patient the familial status was missing, leaving 305 cases with sporadic MD. In Table 3 baseline characteristics of sporadic MD patients in the current study are given and compared to those of the other studies.

**Table 3:** Characteristics of unilateral sporadic cases in the current study compared to previous studies

	Current study	Frejo et a.	*P*-value	Crossley et al.	*P*-value	Chen et al.	*P*-value
Cases, *n*	306	852		68		245	
Gender, n (% women)	152 (49.8)	478 (56.1)	0.059	37 (49.3)	0.495	150 (61.2)	0.008*
Age of onset, mean (SD)	54.3 (14.6)	45.6 (12.5)	< 0.001*	N/A		45.9 (12.7)	< 0.001*
Age of onset ≤ 40, n (%)	57 (18.7)	306 (36.0)	< 0.001*	N/A		80 (32.7)	< 0.001*
Hearing loss at diagnosis, mean (SD)	52.5 (20.1)	50.9 (17.8)	0.258	N/A		54.4 (21.3)	0.220
Delayed MD (%)	71 (23.3)	61 (7.5)	< 0.001*	14 (18.7)	0.633	62 (25.3)	0.305
Migraine, n (%)	55 (18.0)	149 (18.0)	0.848	18 (24.0)	0.113	37 (15.1)	0.360
Autoimmune disease, n (%)	48 (15.7)	146 (19.4)	0.575	14 (18.7)	0.331	6 (2.4)	< 0.001*
Hearing stage, n (%)				N/A			
1	33 (10.8)	64 (7.8)	0.0737			25 (10.2)	0.815
2	60 (19.7)	171 (20.9)	0.881			47 (19.2)	0.886
3	154 (50.5)	430 (52.5)	0.995			126 (51.4)	0.827
4	58 (19.0)	154 (18.8)	0.715			47 (19.2)	0.960
Cardiovascular risk							
High blood pressure, n (%)	117 (38.4)	286 (34.2)	0.132	24 (32.0)	0.637	34 (13.9)	< 0.001*
Dyslipidemia, n (%)	91 (29.8)	307 (37.2)	0.051	26 (34.7)	0.177	38 (15.5)	< 0.001*
Type 2 diabetes, n (%)	23 (7.5)	84 (10.1)	0.230	5 (6.7)	0.958	14 (5.7)	0.395
Smoking, n (%)	43 (14.1)	185 (22.3)	0.004	28 (37.3)	< 0.001*	42 (17.1)	0.326
Tumarkin crisis, n (%)	68 (22.3)	150 (18.1)	0.072	7(9.3)	0.026*	11 (4.5)	< 0.001*

Sporadic population with familial cases excluded for Frejo et al., Crossley et al. and current study. * Statistically significant finding, *N/A* not applicable, *SD* standard deviation, *MD* Menière’s disease. Chi-square and t-tests were used to calculate p-values

Table 4 presents a comparison of characteristics of unilateral MD across the subgroups in the current study. Statistically significant differences in gender were observed between the classic subgroup (47% women) and the autoimmune subgroup (79.2% women), as well as between the delayed subgroup (40.5% women) and the autoimmune subgroup (79.2% women), with an overall p-value of 0.003. In addition, the mean age of onset was significantly higher in the delayed subgroup (59.3 years, SD = 13.9) compared to the classic subgroup (52.9 years, SD = 15.0, *p* = 0.012).

**Table 4:** Characteristics of unilateral Menière’s disease comparing the different subtypes

	Classic MD	Delayed MD	Familial MD	Migraine MD	Autoimmune MD	*P*-value
Cases, *n*	164	79	22	46	24	
Gender, n (% women)	77 (47.0) ˚ꝉ	32 (40.5)˚	12 (54.4)	30 (65.2)	19 (79.2) ˚ꝉ ˚	0.003*
Age of onset, mean (SD)	52.9 (15.0) ꝉ	59.3 (13.9) ꝉ	55.7 (12.9)	52.2 (14.4)	54.42 (13.3)	0.002*
Age of onset ≤ 40, n (%)	37 (22.6)	7 (8.9)	2 (9.1)	11 (23.9)	4 (16.7)	0.061
Hearing loss at diagnosis, mean (SD)	51.4 (20.9)	55.2 (18.3)	55.5 (18.2)	49.2 (18.1)	55.4 (21.5)	0.382
Hearing stage, n (%)						
1	23 (14.0)	6 (7.6)	1 (4.5)	2 (4.3)	2 (8.3)	0.251
2	32 (19.5)	10 (12.7)	5 (22.7)	15 (32.6)	5 (20.8)	0.251
3	79 (48.2)	48 (60.8)	10 (45.5)	22 (47.8)	11 (45.8)	0.251
4	30 (18.3)	15 (19.0)	6 (27.3)	7 (15.2)	6 (25.0)	0.251
Cardiovascular risk						
Mean, BMI (SD)	25.4 (6.8)	25.5 (7.6)	27.4 (4.5)	26.3 (5.8)	24.1 (6.7)	0.506
High blood pressure, n (%)	52 (31.7)	37 (46.8)	5 (22.7)	20 (43.5)	11 (45.8)	0.067
Dyslipidemia, n (%)	47 (28.7)	27 (34.2)	10 (45.5)	9 (19.6)	10 (41.7)	0.133
Type 2 diabetes, n (%)	10 (6.1)	8 (10.1)	1 (4.5)	5 (10.9)		0.613
Smoking, n (%)	21 (12.8)	11 (13.9)	1 (4.5)	9 (19.6)	4 (16.7)	0.527
Tumarkin crisis, n (%)	35 (21.3)	13 (16.5)	3 (13.6)	15 (32.6)	6 (25)	0.612
Anxiety/Depression, n (%)	34 (20.7)	12 (15.2)	2 (9.1)	16 (34.8)	7 (29.2)	0.047∞

Characteristics of unilateral Menière’s disease of current cohort comparing different subtypes * = Statistically significant finding; ∞ no statistical difference was found between cells after Bonferroni correction, ˚ꝉ ˚ statistical difference was found between cells after Bonferroni correction, ˚ꝉ ˚ statistical difference was found between cells marked with the same symbol, *SD* standard deviation, *MD* Menière’s disease. ANOVA and Chi-square and t-tests were used to calculate p-values

In Table 5, the distribution of unilateral MD subtypes is shown, along with the proportion of patients in each group showing overlap with the characteristics of other subtypes.

**Table 5:** Distribution of the subtypes within the unilateral Menière’s disease cohort

	Type 1 (classic MD)	Type 2 (Delayed MD)	Type 3 (Familial MD)		Type 4 (Migraine)	Type 5 (autoimmune MD)
Classic MD, *n* (%)	163 (100)	0		0	0	0
Delayed MD, n (%)	0	79 (100)		0	0	0
Familial, n (%)	0	7 (8.9)		22 (100)	0	0
Migraine, n (%)	0	9 (11.4)		6 (27.3)	46 (100)	0
Autoimmune disease, n (%)	0	14 (17.7)		5 (22.7)	10 (21.7)	24 (100)

Distribution of the subtypes within the unilateral Menière’s disease cohort in the current study. Each row represents patients classified in one subtype, while columns indicate the proportion of those patients exhibiting characteristics overlapping with other subtypes. *MD* Menière’s Disease

## Discussion

In this study, we identified the clinical subtypes of MD as described by Frejo et al. within a Dutch MD patient population and compared subtype distribution to findings from previous studies by Frejo et al., Crossley et al., and Chen et al. [6, 8, 9]. The clinical subtypes identified in our study closely align with those reported by Crossley et al., with no significant differences observed between the two cohorts. However, it is important to consider that the American cohort examined by Crossley et al. was relatively small, potentially limiting its generalizability to the broader U.S. population [8].

When comparing the proportions of patients per subgroup between the different cohorts, the most pronounced difference was found in the unilateral delayed MD subtype, 23.6% in our study versus 8.4% in Frejo’s population [6]. Similarly, the American and Chinese cohorts demonstrated higher rates as well, at 20.8% and 25.3% respectively. These differences may reflect population-specific factors that contribute to distinct clinical manifestations such as genetic variants associated with this phenotype, dietary habits, stress-related influences, or differences in clinical diagnostic practices [8, 9].

In our study, 6.6% of patients were classified in the familial MD subtype, which is significantly lower than the 12.7% reported by Frejo et al. but significantly higher than the 2.4% in the Chinese cohort by Chen et al. [6, 9]. This likely reflects geographic differences in MD prevalence, suggesting a stronger role of genetic predisposition in European and North American populations compared to Asian populations [11]. The proportion of familial cases in Crossley et al. was similar to ours, whereas Frejo et al. reported a higher rate of familial MD, possibly reflecting an overrepresentation of familial cases in their Spanish cohort due to the research group’s focus on genetic variants of MD [6, 8]. Table 5 shows that not all familial cases are included in the familial MD cohort, as some fall under the delayed MD subtype, the subtype that had a significant lower representation in Frejo’s study [6]. Therefore, in this study, the overall proportion of MD patients with an affected family member was 8.8%.

No differences were observed in the distribution of the migraine MD subtype between our study and the other three studies [6, 8, 9]. A large-scale study previously reported a migraine prevalence of 10% among MD patients, compared to 3.5% in a matched control population without MD [12]. Our study found an even higher prevalence of 18.1% within the entire MD cohort. This supports earlier observations of a potential association between migraine and MD.

In the current study 7% of the unilateral MD cohort was classified under the autoimmune MD subtype, which aligns with findings from the other studies [6, 8, 9]. Overall, 15.7% of unilateral MD patients had an autoimmune disease, which was significantly higher than the 2.4% in the Chinese cohort [9]. Autoimmune diseases are known to be more prevalent in Europe (10.2%) than in Asia (3%) [13, 14]. A recent systematic review and meta-analysis examining the association between MD and immune-related disorders also reported regional differences, particularly between European and Asian populations [15]. While these observations suggest that environmental and lifestyle factors contribute to susceptibility, genetic differences between populations may also partly explain the observed variation.

Comparison of bilateral subtypes revealed significant differences in the synchronic MD subgroup between our study and of the study of Frejo et al. (Fig. 1) [7]. Given the relatively small sample size of our bilateral MD cohort and the widely reported prevalence of bilateral MD (2–78%), these findings should be interpreted with caution [16].

Comparison of MD subtypes across studies may offer insights into population differences, but it has to be taken into account that studies differed as to approaches to data collection and subtype classification. In our study, most patients were contacted directly, whereas other studies primarily relied on medical records, which may be incomplete or outdated [6, 8]. In both our study and others, the retrospective nature creates a risk of recall bias with regards to subgroup classification, and criteria used to define specific subtypes were not always clearly described. These inconsistencies underscore the potential challenges in defining and comparing subgroups across the different studies.

When examining the differences in characteristics between the clinical subtypes in our cohort, represented in Table 4, significant differences were observed in gender and age of onset. The gender difference may be attributed to the higher prevalence of autoimmune diseases among women [13]. The variation in age of onset could reflect a characteristic feature of this particular subtype; however, in the other studies this difference was not mentioned or not observed. Additionally, for unilateral sporadic cases, our study reported a significantly higher mean age of onset (54.3 years) compared to the findings of Frejo et al. (45.6 years) and Chen et al. (45.9 years) [6, 9]. Similarly, the proportion of patients with disease onset before the age of 40 was significantly higher in the Spanish (36%) and American (32.7%) cohorts compared to our cohort (18.7%) [6, 8]. This variation may be influenced by genetic, environmental, or lifestyle differences across cohorts. Interestingly, a study by van Esch et al. conducted in the Netherlands reported a comparable mean age of onset of 53 years, aligning with our findings [17]. We have verified that this study involved a different cohort, ensuring that the results are not directly comparable.

In terms of smoking, only 14.1% of patients in our Dutch cohort were current or former smokers (the latter defined as having quit smoking within the past 15 years), which was significantly lower than in the Spanish (22.3%) and American (37.2%) cohorts [6, 8]. This difference in smoking rates between Dutch and Spanish MD patients may simply reflect the general differences in smoking prevalence between these countries, without implying any causal effect of smoking on MD.

Foster and Breeze proposed the theory that endolymphatic hydrops, combined with cardiovascular risk factors, may intermittently reduce inner ear perfusion, contributing to the development of MD [18]. If so, risk factors would be more common in MD patients. However, prevalence rates in the general population of 29.4% for hypertension, 34.1% for dyslipidemia, and 12% for diabetes, are similar to those in our cohort (38.4%, 29.8%, and 7.5%, respectively), as well as in the populations studied by Frejo et al. (34.2%, 37.2%, and 10.1%) and Crossley et al. (32.0%, 34.7%, and 6.7%) [6, 8, 19]. When comparing our population to that of Chen et al., significant differences are found in hypertension (13.9%) and dyslipidaemia (15.5%) [9]. This variation underscores the wide global differences in cardiovascular risk factor prevalence.

An interesting finding in our study is the significantly higher prevalence of Tumarkin drop attacks in our study (22.3%) compared to Crossley et al. (9.3%) and Chen et al. (4.5%) [8, 9]. This difference may be due to differences in cohort composition: Chen et al. included only patients diagnosed recently, whereas our study included patients with a longstanding diagnosis of MD [8, 9]. Since drop attacks typically occur in later stages of the disease, the higher prevalence is likely a reflection of the inclusion of MD cases with a long disease duration [20]. A recent systematic review on vestibular drop attacks showed prevalence estimates ranging from 3 to 72%, possibly due to the lack of a definite definition of a drop attack [20]. which also may have been of influence in the comparison made between the above mentioned studies and our cohort.

Clearly, there are certain limitations of the present study, such as a potentially high recall bias and occasional difficulty in establishing a definitive diagnosis needed for accurate subtyping. While recalling patients may not completely eliminate bias, especially depending on the time elapsed since symptom onset, our study contacted almost all patients individually to retrieve missing information, enhancing completeness of the dataset and more consistency in diagnosis. Furthermore, we investigated a large and representative sample of the Dutch MD population.

Distinguishing between subtypes of MD in future clinical trials may help clarify why certain interventions are effective in some patients but not in others. Given that MD development likely depends on multiple factors including genetic, lifestyle, and environmental influences, subtype-specific analyses could reveal differential treatment responses and improve the interpretation of clinical trial results, supporting the development of more individualized treatment approaches, ultimately improving patient outcomes.

In conclusion, the subtype classification proposed by Frejo et al. proved to be applicable in a large Dutch cohort. Notable geographic differences in subtype distribution, particularly in the prevalence of delayed MD and familial MD, may reflect population-specific influences such as genetic and environmental factors in the development and presentation of MD.
